# The Effect of Mold Structure and Cooling Parameters on Heat Transfer during Billet High-Speed Continuous Casting

**DOI:** 10.3390/ma16093361

**Published:** 2023-04-25

**Authors:** Sijie Wang, Pei Xu, Yongzhi Zhou, Huamei Duan, Dengfu Chen, Mujun Long

**Affiliations:** Laboratory of Materials and Metallurgy, College of Materials Science and Engineering, Chongqing University, Chongqing 400044, China

**Keywords:** billet continuous casting, high-speed casting, mold, heat transfer

## Abstract

Mold structure and cooling parameters are significant factors that affect the heat transfer capacity of high-speed continuous casting molds of billets. Therefore, a three-dimensional fluid flow and heat transfer model of a 160 mm × 160 mm billet mold was established, and its accuracy was verified. Thereby, the characteristics of heat transfer and influences of mold structure and cooling parameters on heat transfer in the high-speed continuous casting billet mold region were revealed. It was found that extending the effective length of a mold is the most valuable method to improve its heat transfer capability and achieve high-speed continuous casting. The total heat and the shell thickness at a mold outlet increased by 19% and 9.21% on average with every 100 mm extension. Enlarging the fillet radius could enhance the uniformity of heat transfer in the mold. Considering the loss of material, the optimal fillet radius of the mold was determined to be R = 10 mm.

## 1. Introduction

To further reduce costs, boost efficiency and realize headless rolling, the high-speed continuous casting of billets has always been the goal of metallurgical workers and steel enterprises [1,2]. In the conventional production of the continuous casting of billets, the casting speed usually ranges between 3 and 4 m/min. Nonetheless, advanced high-speed casting techniques have enabled the attainment of significantly faster rates, reaching 6.5 m/min and even surpassing this. [3]. The high-speed continuous casting of billets is a systematic project that encompasses a range of technologies, including steel ladle technology, tundish technology, mold technology, secondary-cooling technology, etc. Of these, the mold technology, which is the heart of the caster, is crucial for the realization of high-speed casting.

However, compared to conventional casting speeds, the heat flux in the billet mold significantly increases and the solidification time of molten steel is shortened under a high casting speed, resulting in a reduction in the solidified shell thickness and its poor growth uniformity, leading to billet cracks and steel leakage accidents [4]. To achieve high-speed continuous casting, it is necessary to ensure efficient heat transfer in the mold area to avoid steel leakage due to a thin shell thickness at the outlet. The structure and cooling parameters of a water tube billet mold have a significant impact on its heat transfer ability. Therefore, on the basis of clarifying the heat transfer behavior of the billet mold, optimizing its structure and cooling parameters is of great theoretical significance to enhance its heat transfer ability and achieve the high-speed continuous casting of billets.

To clarify the heat transfer phenomenon in the mold region, Mizikar et al. [5] and Lait et al. [6] developed a one-dimensional mathematical model to acquire the temperature distribution of a shell, which paved the way for the mathematical simulation of heat transfer behavior in the mold region. However, due to the restricted conditions, early simulation studies on molds were oversimplified and did not fully represent reality. Thanks to the improvement in computer processing power and the evolution of simulation software, more comprehensive variables and precise computations of three-dimensional mathematical models [7,8,9] have been implemented to scrutinize heat transfer behavior in molds. These models play a crucial role in optimizing the molds’ parameters.

Optimizing the structure of a mold and the cooling parameters are important methods to enhance heat transfer capability in the mold region. As the container and medium for the heat transfer of molten steel, the structure of copper tubes has a significant impact on the cooling of molten steel. Yang et al. [10] investigated the effect of different copper tube thicknesses on the temperature distribution of the copper tube’s thermal surface in a slab mold and found that a thinner copper tube resulted in a lower temperature. Yu et al. and Xie et al. [11,12] studied the difference in temperature field, solidification shell thickness, and heat flux distribution of molten steel on slab molds with different fillet structures. They suggested that a right-angle mold had the best heat transfer effect but the most uneven temperature distribution on the surface of the shell. The length of the copper tube largely determines the residence time of molten steel in a mold, but currently, there is no detailed research on its impact on the heat transfer of a mold.

The heat from molten steel in a mold is ultimately dissipated by cooling water. Therefore, it is important to study the influence of cooling parameters on the heat transfer capability of a mold. Chow et al. [13] established a mathematical model for a 120 mm × 120 mm billet continuous casting mold, a copper tube thickness of 14 mm and a mold length of 1000 mm, and they found that the effect of cooling water velocity on heat transfer behavior in the mold was small. Wu et al. [14] established a three-dimensional flow and heat transfer model for the mold region of a 140 mm × 140 mm billet, a copper tube thickness of 10 mm, a mold length of 1000 mm, a water gap width of 4.0 mm and a casting speed of 2.2 m/min. The simulation analyzed the temperature field, solidification shell thickness and copper tube cold surface temperature in the mold under a cooling water velocity of 11.70~13.17 m/s. The results showed that adjusting the velocity of cooling water was beneficial to avoiding the billet rhomboidity phenomenon. Yang et al. [10] studied the effect of different cooling water velocities on the temperature distribution of a copper tube’s hot surface and pointed out that the effect of water velocity on temperature weakened when the water velocity exceeded 8.0 m/s. Water gap width, as the main parameter of a mold’s water-cooling structure, has not been studied in detail regarding its impact on heat transfer in molds.

After analyzing the current state of research on heat transfer in billet molds, it can be concluded that most studies have focused on conventional casting speed [7,13,14,15,16], and the impact of a mold’s structure and cooling parameters [10,12,13,14] has not been systematically studied. Therefore, this paper presents a three-dimensional fluid flow and heat transfer model of a 160 mm × 160 mm billet mold, consisting of molten steel, flux film, copper tube and cooling water. Based on this model, the characteristics of heat transfer and influences of a mold’s structure and cooling parameters, such as the effective length of mold, the width of the copper tube, the velocity of the cooling water, etc., on heat transfer in the high-speed (6.5 m/min) continuous casting billet mold region was revealed, and key influencing parameters were determined. It is significant to acknowledge that this study has some limitations, primarily stemming from model assumptions, the determination of material parameters, the validation of simulation results, and other factors, which may have led to the calculation results deviating from reality. However, the importance of this paper lies in its contribution to laying the essential theoretical and technical foundation for the industrialization of high-speed billet continuous casting by studying the heat transfer of the core component (mold) of the caster.

## 2. Model Description

### 2.1. Assumptions

To facilitate the subsequent study on heat transfer in a mold, this paper put forward the following reasonable assumptions when establishing the mathematical model:

(1) Liquid steel and water are uncompressible Newtonian fluid.
(2) The oscillation of the mold and its arc structure are neglected.
(3) Due to its minimal thickness, the mold coating is ignored.
(4) The shrinkage of the solidified shell is ignored.
(5) Based on the symmetry of the billet mold, the 1/4 model is established.

### 2.2. Geometric Model

**Figure 1:**
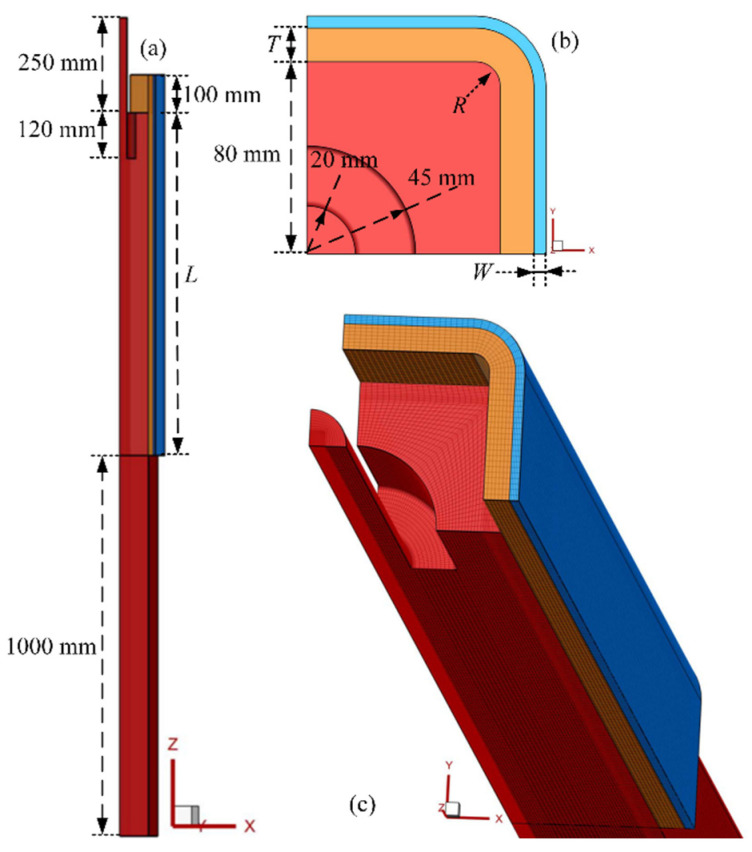
Geometrical model and meshing of billet’s continuous casting mold region: (**a**) front view, (**b**) top view and (**c**) meshing.

Based on the symmetry of the research object, a three-dimensional geometry model of 1/4 solidification and heat transfer was established, which integrated molten steel, a submerged nozzle, flux film, a copper tube and cooling water, as shown in Figure 1a,b. The reference parameters in Figure 1a,b are shown in Table 1. To prevent interference from the backflow that occurred at the mold outlet, a section of the molten steel was extended by 1000 mm, as illustrated in Figure 1a. The geometry model was then meshed using Ansys ICEM, as seen in Figure 1c. The maximum mesh size for the molten steel in the mold was set at 4 mm, while the extension section was set at 5 mm. The mold copper tube and cooling water were meshed with sizes of 2 mm and 1 mm, respectively. Furthermore, the local grid size of the 10 mm thickness, located away from the strand surface, was refined to 1 mm in the circumferential direction of the billet. In the process of numerical simulation, the commercial computational fluid dynamics software Fluent 19.2 was used.

### 2.3. Physical Properties

The materials incorporated into this model comprised molten steel, copper and water. The physical properties of them are listed in Table 2 and Table 3.

### 2.4. Governing Equations

The governing equations utilized in this study were referenced from the research of Wu et al. [14] and Chen et al. [18], as detailed below.

Energy equation

(1) $$\frac{\partial }{\partial t}(\rho H)+\nabla \cdot (\vec{v}(\rho H+P))=\nabla \cdot (k_{eff}\nabla T)+S$$

where H is enthalpy, which can be calculated by Equation (2); $k_{eff}$ is effective thermal conductivity; S is the source term, which represents the internal heat source in the fluid and the portion of mechanical energy converted into thermal energy due to viscosity and is taken as zero in this study.

(2) $$H=h_{ref}+\int_{T_{ref}}^{T}C_{p}dT+\beta L$$

Continuity equation

(3) $$\frac{\partial }{\partial t}+\nabla (\rho \vec{\nu })=0$$

Momentum equation (Navier–Stokes equation)

(4) $$\rho \frac{\partial \vec{v}}{\partial t}+\rho \nabla \cdot (\vec{v}\vec{v})=-\nabla P+\nabla \cdot (\vec{\bar{\tau }})+\rho \vec{g}+\vec{F}$$

(5) $$\vec{\bar{\tau }}=\mu \left[\left(\nabla \cdot \vec{v}+\nabla \cdot {\vec{v}}^{T}\right)-\frac{2}{3}\nabla \vec{v}I\right]$$

where *P* denotes the static pressure, $\vec{\bar{\tau }}$ represents the stress tensor, and $\rho \vec{g}$ and $\vec{F}$ are the gravitational body forces and external body forces, respectively.μ is the molecular viscosity, and I is the unit tensor.

Considering the momentum sink caused by the formation of mushy zone during solidification, the source term in Equation (4) was added, as follows:

(6) $$S_{m\;}=\vec{F}=\frac{{\left(1-\beta \right)}^{2}}{\left(\beta^{3}+0.001\right)}A_{mush\;}\left(\vec{v}-{\vec{v}}_{pull}\right)$$

where β represents the liquid volume fraction, ${\vec{v}}_{pull}$ represents the casting speed, $A_{mush\;}$ represents the mushy zone constant, generally between 10^5^ and 10^8^ [19], and the value of this model is 10^8^.

k−ε equations

The impact of turbulence is explained using standard k−ε equations. The details are as follows:

(7) $$\frac{\partial }{\partial t}(\rho k)+\frac{\partial }{\partial x_{i}}\left(\rho kv_{i}\right)=\frac{\partial }{\partial x_{j}}\left[\left(\mu +\frac{\mu_{t}}{\sigma_{k}}\right)\frac{\partial k}{\partial x_{j}}\right]+G_{k}+G_{b}-\rho \varepsilon -Y_{M}+S_{k}$$

(8) $$\frac{\partial }{\partial t}(\rho \varepsilon )+\frac{\partial }{\partial x_{i}}\left(\rho \varepsilon v_{i}\right)=\frac{\partial }{\partial x_{j}}\left[\left(\mu +\frac{\mu_{t}}{\sigma_{\varepsilon }}\right)\frac{\partial \varepsilon }{\partial x_{j}}\right]+C_{1\varepsilon }\frac{\varepsilon }{k}\left(G_{k}+C_{3\varepsilon }G_{b}\right)-C_{2\varepsilon }\rho \frac{\varepsilon^{2}}{k}+S_{\varepsilon }$$

where $G_{k}$ and $G_{b}$ are the generation of turbulence kinetic energy due to the mean velocity gradients and buoyancy, respectively. $Y_{M}$ is the contribution of the fluctuating dilatation in compressible turbulence to the overall dissipation rate. $C_{1\varepsilon }$, $C_{2\varepsilon }$, $C_{3\varepsilon }$, $\sigma_{k}$ and $\sigma_{\varepsilon }$ are constants of this model. $S_{k}$ and $S_{\varepsilon }$ are the source terms, similar to $S_{m\;}$.

The calculation formula for $\mu_{t}$ is as follows:

(9) $$\mu_{t}=\rho C_{\mu }\frac{k^{2}}{\varepsilon }$$

The model constants $C_{1\varepsilon }$, $C_{2\varepsilon }$, $C_{3\varepsilon }$, $C_{\mu }$, $\sigma_{k}$ and $\sigma_{\varepsilon }$ are empirical parameters. The values taken in this study are as follows: $C_{1\varepsilon }=1.44$, $C_{2\varepsilon }=1.92$, $C_{3\varepsilon }=C_{\mu }=0.09$, $\sigma_{k}=1.00$ and $\sigma_{\varepsilon }=1.30$.

### 2.5. Boundary Conditions

Momentum boundary conditions

The velocity-inlet boundary condition was employed at the molten steel inlet, and the velocity was calculated using the mass conservation equation, as depicted in Equation (10). The inlet boundary condition of cooling water was similar to this. At the outlet, both of them were set up as the pressure outlet [20]. A specified shear and thermal isolation were applied to the surface of the molten steel in the billet mold.

(10) $$v_{inlet}=v_{casting}\cdot \frac{S_{inlet}}{S_{outlet}}$$

where $v_{inlet}$ is the inlet velocity of SEN, and $v_{casting}$ is the casting speed. $S_{inlet}$ is the inlet area of SEN, and $S_{outlet}$ is the outlet area of mold.

Heat transfer boundary conditions

The temperature of molten steel and cooling water at the inlet were 1808 K (pouring temperature) and 300 K. At the outlet, both of them were set up as the pressure outlet [20]. The molten steel surface in the billet mold was isolated with specific shear and thermal conditions. The interfaces of the molten steel/hot surface of the mold copper tube and the cold surface of the mold copper tube/cooling water were both subjected to a coupled interface. The comprehensive thermal resistance that accounted for the impact of the mold flux film and air gap was loaded into the coupled interface through a user-defined function (UDF) [11].

Heat transfer process in the mold area

**Figure 2:**
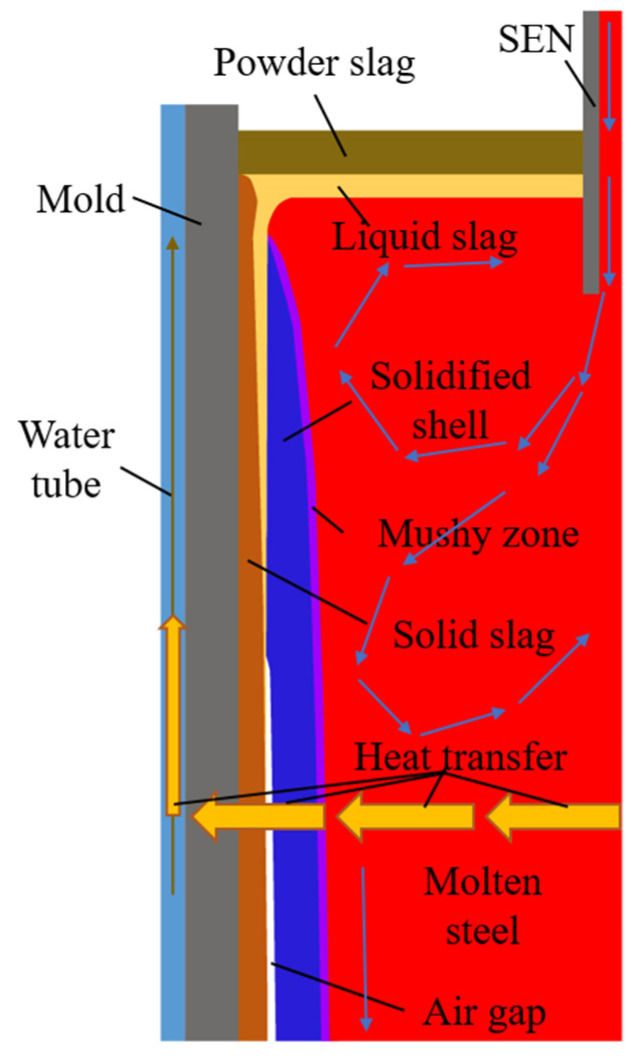
Schematic diagram of heat transfer in mold region.

Figure 2 shows the heat transfer process of molten steel in the billet’s continuous casting mold area. The heat of molten steel injected into the mold through the SEN passed through the mushy zone, solidified shell, mold flux film, and copper tube, and was ultimately carried away by the cooling water.

### 2.6. Model Verification

Due to the limited research on heat transfer in billet molds under high casting speeds, it was difficult to verify the results at 6.5 m/min. Therefore, in this paper, the simulation results of the model under 3.0 m/min casting speed were compared and analyzed with existing literature data and measurement values (with similar casting speeds and billet sections). The heat flux distribution and the variation in shell thickness along the casting direction at the centerline of the shell surface were the key results used for validation. The details are described here.

**Figure 3:**
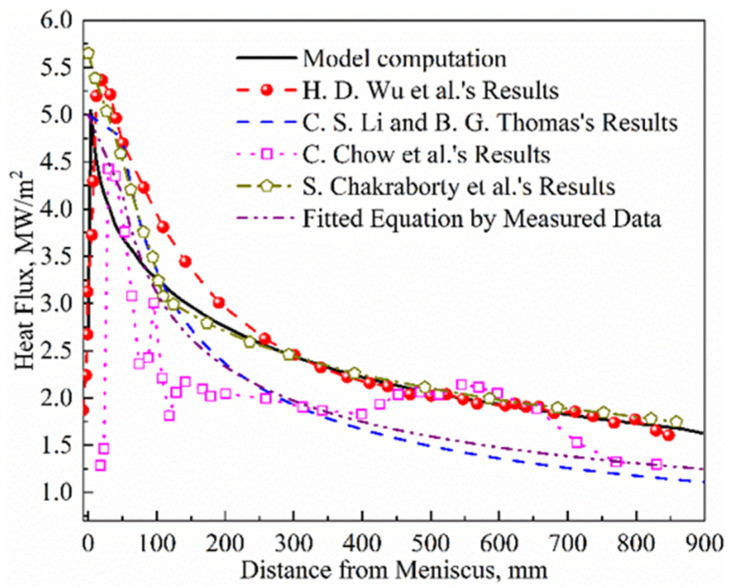
Comparison of heat flux distribution in mold. Model validation data from references [14,21,22,23].

Figure 3 shows the heat flux along the casting direction at the centerline of the shell surface. The results show that the variation in heat flux in this model is generally consistent with that in other literature and actual measurements. These curves generally rise sharply from the meniscus and reach a peak of about 5.0–5.7 MW/m^2^ about 20–50 mm below the meniscus; then, within a range of about 50–300 mm from the meniscus, the heat flux rapidly decreases. After the heat flux decreases to a range of 2.0–2.5 MW/m^2^, the decreasing trend begins to level off, until the outlet of the mold is reached.

**Figure 4:**
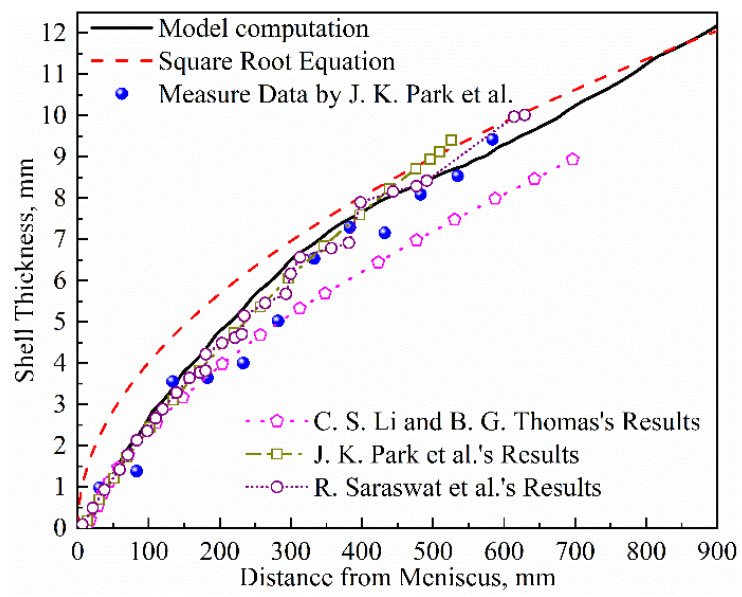
Comparison of solidified shell thickness in mold. Model validation data from references [20,24,25].

Figure 4 shows shell thickness along the casting direction at the centerline of the shell surface. The results demonstrate that the calculated shell thickness obtained by the model is in good agreement with the results obtained by different researchers. The shell thicknesses at the mold outlet range from 8 mm to 12 mm. In the upper region of the mold, the shell thicknesses increase rapidly, while the solidification rates of the molten steel decrease in the middle and lower region of the mold.

Although the specific data in the aforementioned comparisons differ slightly due to variations in cross-sections and casting speeds between different sources and this study, the overall trends are consistent. This indirectly validates the accuracy of the model used in this study.

## 3. Results and Discussion 

### 3.1. Characteristics of Heat Transfer under High Casting Speed

Before investigating the effects of different mold structures and cooling parameters on heat transfer in the billet mold under a high casting speed (6.5 m/min), this paper conducted a detailed comparative analysis of heat transfer behavior in the mold under different casting speeds to clarify the heat transfer characteristics in the mold under a casting speed of 6.5 m/min, laying the foundation for subsequent research.

**Figure 5:**
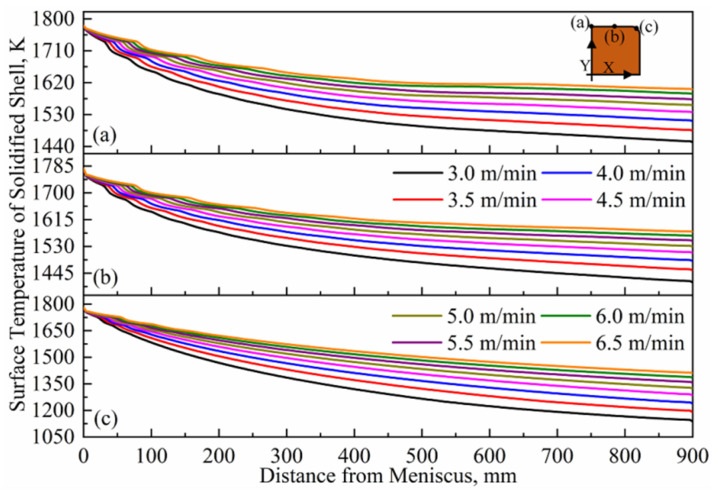
Temperature distribution on characteristic position of solidified shell’s surface at different casting speeds: (**a**) center, (**b**) 1/4 and (**c**) corner.

Temperature distributions at three characteristic positions (center, 1/4 and corner) under different casting speeds are plotted in Figure 5. It can be seen that the temperature changes have a consistent trend. With an increasing casting speed, the temperature at the three characteristic positions shows an upward trend, but the magnitude of the increase gradually decreases. Compared with the casting speed of 3.0 m/min, the temperatures of the mold exit at the three characteristic positions increase from 1452 K to 1601 K, 1416 K to 1577 K and 1138 K to 1410 K, respectively, with an increase rate of 10.26%, 11.37% and 23.90%. In the circumferential direction of the billet, the temperature difference between the center and corner at the mold outlet decreases from 314 K to 191 K. This indicates that a high casting speed not only significantly increases the surface temperature of billets, but also contributes to the uniformity of heat transfer in a mold.

**Figure 6:**
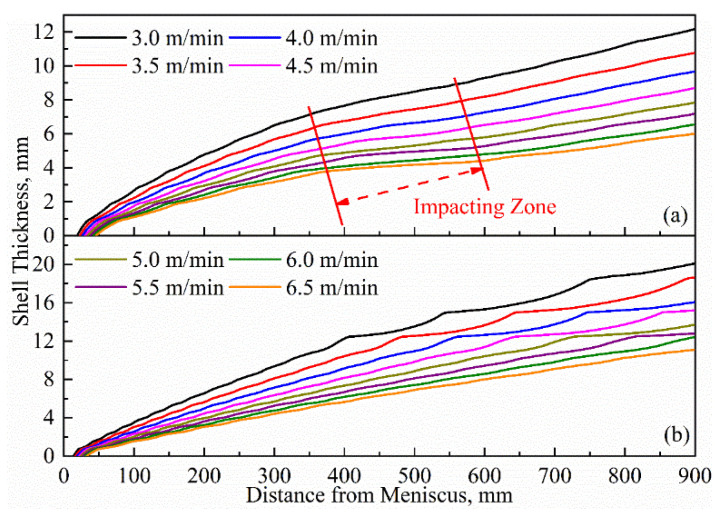
Variation in shell thickness under different casting speeds: (**a**) center and (**b**) corner of the strand.

The variation in shell thickness can indicate the magnitude of the heat transfer capacity of the mold. Figure 6 shows the variation in shell thicknesses under different casting speeds at the center and corner of billet. The thicknesses of the shells grow fastest in the initial solidification stage, but they slow down due to the remelting of the solidification front, caused by the impact of high-temperature molten steel backflow in the range of 350–600 mm from the meniscus. In the later stage of the mold, compared with the 3.0 m/min casting speed, the growth curve of the shell thickness at the corner is significantly smoother at the 6.5 m/min casting speed. This is due to the overall thin shell thickness at the corner under a high casting speed, resulting in the growth being less affected by the impact of molten steel backflow. Compared with 3.0 m/min, the shell thicknesses at the two characteristic positions decrease significantly at the 6.5 m/min casting speed, from 12.18 mm to 6.01 mm at the center and from 20.11 mm to 10.96 mm at the corner, with a decrease rate of 50.66% and 45.50%, respectively. This will greatly increase the risk of steel leakage, and it also indicates that to achieve the high-speed continuous casting of billets, the heat transfer capacity of the mold region must be improved.

### 3.2. Effect of Mold’s Effective Length

Extending the effective length of a mold is one of the most valuable methods to solve the problem of insufficient solidification times of molten steel under high casting speeds. In existing studies, the maximum mold effective length of the billet designed to increase the casting speed was 1400 mm. Therefore, this study investigated heat transfer in the mold with its effective length ranging from 900 to 1400 mm.

**Figure 7:**
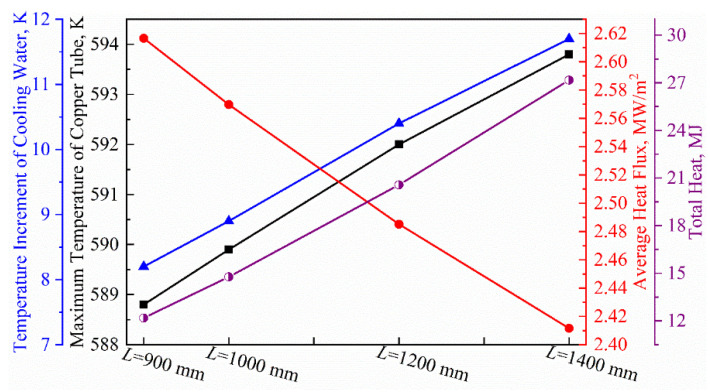
Behavior of heat transfer in billet mold with different effective lengths.

As the mold’s effective length increases from 900 mm to 1400 mm, as shown in Figure 7, the maximum temperature of the copper tube increases from 506 K to 510 K, with an increase rate of 0.79%, which is basically unchanged. The temperature of the cooling water increases from 8.22 K to 12.16 K, with an increase rate of 47.93%. The total heat transfer in the mold region increases from 12.18 MJ to 27.17 MJ, which is growth of approximately 123%. This means that extending the effective length of the mold can significantly improve its heat transfer capacity. Specifically, for every 100 mm increase in effective mold length, the total heat transfer increases by an average of 2.89 MJ, a growth rate of about 19%. However, the average heat flux density decreases linearly from 2.58 MW/m^2^ to 2.42 MW/m^2^, which is a drop of about 8.02%. This is because extending the effective length of the mold not only increases its total heat transfer, but also significantly increases the heat transfer surface area and the residence time of the steel liquid in the mold region. When the increase in these two factors exceeds the increase in total heat transfer, the ratio of total heat transfer to heat transfer surface area and residence time (heat flux) shows a decreasing trend.

**Figure 8:**
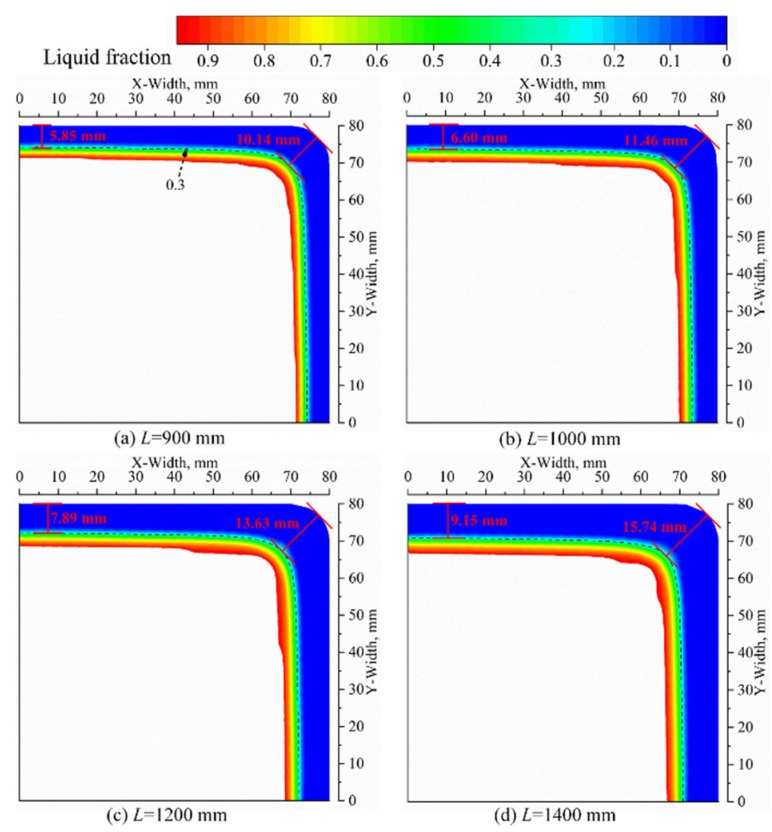
Liquid fraction of the strand at mold outlet with four molds’ effective lengths.

Figure 8 illustrates the solidification shell at the outlet of the mold for various mold lengths. As the mold’s effective length increases from L = 900 mm to L = 1400 mm, the thickness of the shell at the center of the billet surface increases from 5.85 mm to 9.15 mm, corresponding to a growth rate of 56.41%. Similarly, the shell thickness at the corner locations increases from 10.14 mm to 15.74 mm, with a growth rate of 55.23%. On average, for every 100 mm increase in the mold’s effective length, the thickness of the solidification shell at the center and corner locations increases by 0.62 mm and 1.08 mm, respectively, with corresponding growth rates of 9.21% and 8.94%. These findings highlight the significant enhancement in heat transfer capability in the mold region achieved by increasing the mold’s effective length, resulting in a substantial increase in the heat transfer area and residence time of the molten steel.

### 3.3. Effect of Copper Tube Thickness and Water Gap Width

The thickness of copper tube and width of water gap have a significant impact on the efficient heat transfer in the water tube mold. In general, the thickness of copper tube is between 8 and 14 mm, and the width of the water gap is between 3 and 5 mm. Therefore, this paper investigates the effect of these two factors on heat transfer in the mold; the required calculation parameters are shown in Table 4.

**Figure 9:**
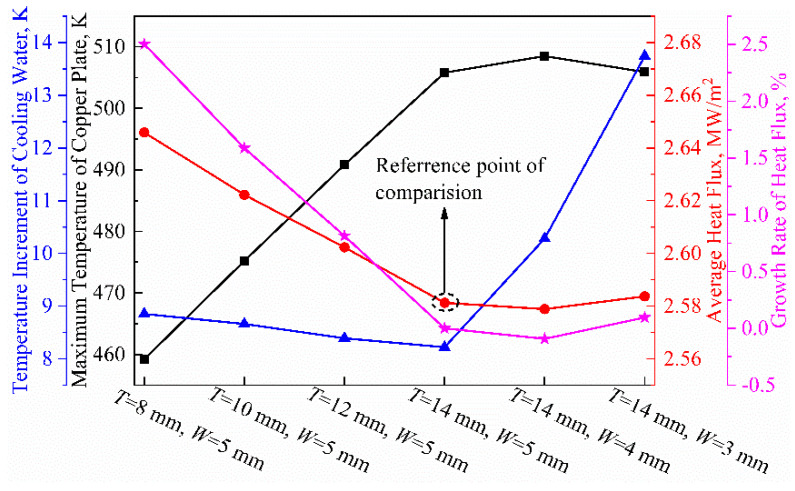
Influence of copper tube thickness and water gap width on heat transfer behavior of mold region.

Figure 9 depicts the effect of the copper tube’s thickness and the water gap’s width on the heat transfer of the mold. The heat transfer capability of the mold improves as the thickness of the copper tube decreases from T = 14 mm to T = 8 mm due to a decrease in thermal resistance. The average heat flux on the surface of the solidified shell increases from 2.56 MW/m^2^ to 2.65 MW/m^2^, which represents a 3.5% increase. The temperature of the cooling water increases from 8.22 K to 8.85 K, representing a change of 7.66%. The highest temperature on the copper tube decreases from 506 K to 459 K, representing a decrease of 9.29%. The thickness of the copper tube in the mold has the greatest impact on the temperature of the copper tube, followed by the temperature of the cooling water, and finally, the overall heat flux.

When the width of water gap decreases from W = 5 mm to W = 3 mm, the average heat flux on the mold is approximately 2.58 MW/m^2^, with a change rate of less than 1%. The highest temperatures of the copper tubes are basically around 589 K, with negligible variation. However, the temperature of the cooling water increases from 8.0 K to 13.7 K, which is an increase of approximately 67.07%, which is a significant change. This is because the reduced width of the water gap leads to a decrease in the flow rate of water passing through the gap in the same amount of time. As a result, the temperature of the cooling water will increase significantly while the heat flux remains basically constant. Therefore, it can be concluded that the temperature rise of the cooling water is extremely sensitive to the width of water gap, but the average heat flux and the highest temperature of the copper tube are not significantly affected.

**Figure 10:**
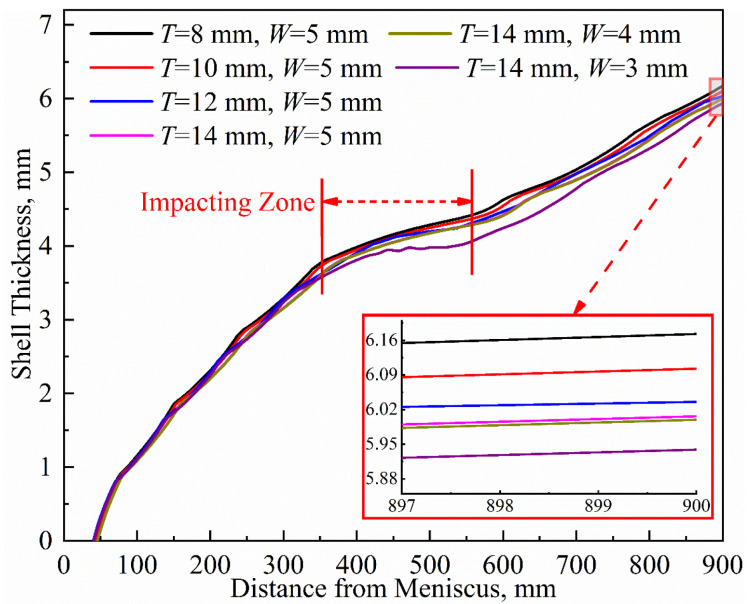
Thicknesses of solidified shell in mold under different parameters.

Figure 10 shows the variations in the thickness of the solidified shell. Similar solidification patterns are observed across different parameters. In the range of approximately 350 to 550 mm away from the meniscus, the impact of high-temperature molten steel causes a re-melting phenomenon at the solidification front, resulting in a slower growth rate of the shell thickness. The shell thickness at the mold outlet is relatively uniform, ranging from 5.94 mm to 6.17 mm under the six parameter combinations.

This study shows that the effect of copper tube thickness on heat transfer in the mold region is higher than that of the water gap width, but overall, the effect is relatively small. The temperature rise of the cooling water is extremely sensitive to the change in the water gap width. This has significance for guidance regarding boiling water under high-speed casting conditions.

### 3.4. Effect of Cooling Water Velocity

Increasing the velocity of cooling water is the most convenient method to enhance the heat transfer capacity of the mold. This study conducted numerical simulations on the heat transfer behavior of the billet’s high-speed continuous casting mold, based on the common cooling water flow velocity of 8–20 m/s [13].

**Figure 11:**
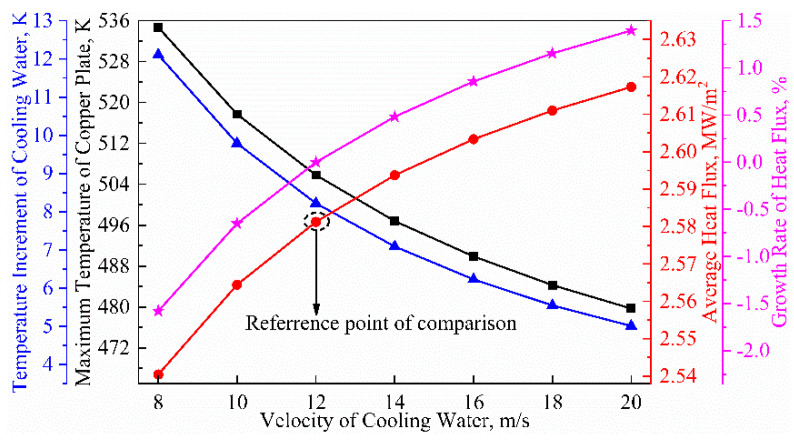
Influence of cooling water’s velocity on heat transfer behavior in billet mold.

Figure 11 illustrates that the average heat flux gradually increases in a “quadratic curve” pattern, with a growth rate of 3.14%, as the velocity of cooling water increases from 8 m/min to 20 m/min. Specifically, the average heat flux rises from 2.54 MW/m^2^ to 2.62 MW/m^2^. However, the cooling water temperature and the maximum temperature of the copper tube show a decreasing trend of 58.63% and 10.29%, respectively, decreasing from 12.11 K to 5.01 K and 534.7 K to 479.7 K. The effect of the cooling water velocity on the shell thickness is found to be small, with the shell thickness of mold outlet ranging from 5.91 mm to 6.11 mm. It was concluded that increasing the velocity of cooling water has the most significant effect on the cooling water temperature rise and helps to prevent the cooling water from boiling in the mold region during high-speed casting.

After conducting research in the three aforementioned parts, it has been determined that the effective length of the mold is the most critical factor that limits its heat transfer capability, followed by the thickness of the copper tube, the cooling water flow rate and the water gap width. While reducing the thickness of the copper tube and increasing the velocity of cooling water can enhance the mold’s heat transfer capability, the extent of this improvement is relatively small. Additionally, an excessively thin thickness of the copper tube can result in the deformation of the mold. Thus, making significant adjustments to the copper tube thickness and cooling water flow rate is not recommended. Consequently, extending the effective length of the mold is the most effective method to increase the mold’s heat transfer capability.

### 3.5. Effect of Fillet Radius

The above research focused on improving the heat transfer capability of the mold and ignored the uniformity of solidification. The fillet radius of the billet’s continuous casting mold is an important means to improve the uniformity of solidified shell. To achieve this, the study investigated the heat transfer behavior of molten steel within the mold with fillet radius ranging from 6 to 25 mm to ascertain the impact on the solidification process.

**Figure 12:**
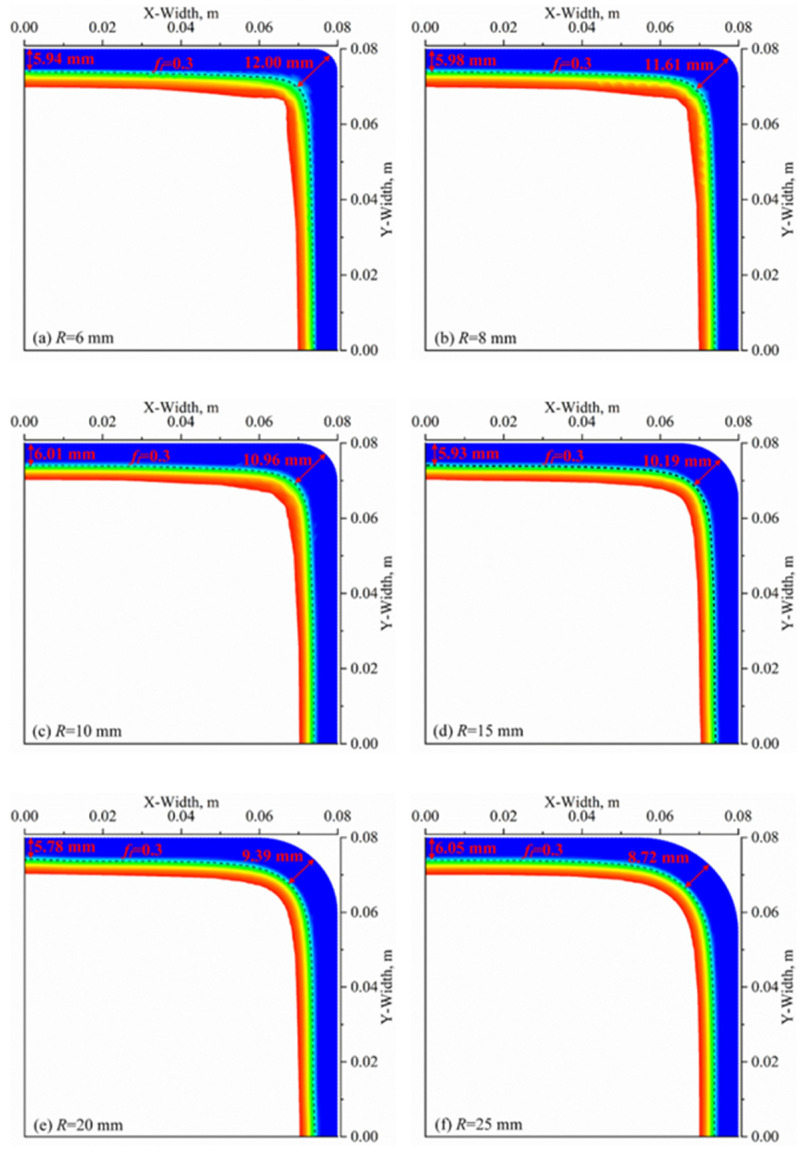
Distributions of solidified shell at mold outlet with different fillet radiuses.

Figure 12 shows the distributions of the solidified shell at the mold outlet with different fillet radiuses. As the fillet radius increases from R = 6 mm to R = 25 mm, the thickness of the shell at the corner gradually decreases from 12.0 mm to 8.72 mm, a decrease of 27.33%, which is significantly reduced, while the one at the center is within the range of 5.78–6.05 mm with a minor variation. This study reveals that the variation in the fillet radius has a notable impact on the heat transfer behavior of the corner, while its impact on the center is negligible. With the increase in the fillet radius, the thickness of the shell at the corner and center gradually approaches each other, indicating an improvement in the heat transfer uniformity in the mold region.

**Figure 13:**
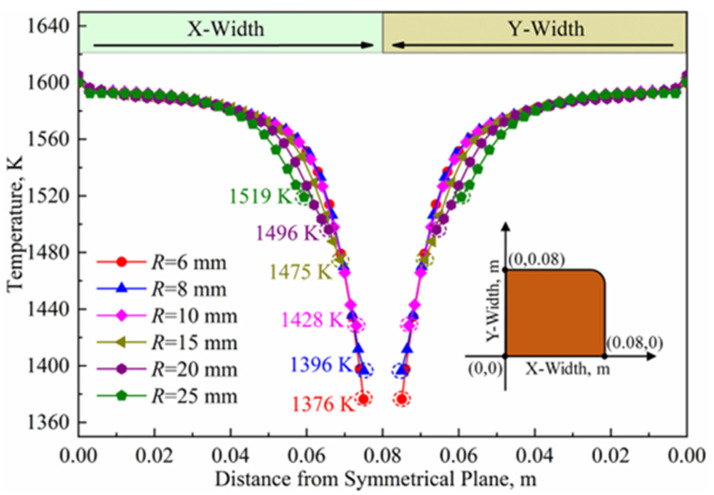
Temperature distributions along circumferential surface of solidified shell at mold outlet.

However, when considering the loss of the billet in actual continuous casting, the fillet radius cannot be too large. Figure 13 illustrates the temperature distributions along the circumferential surface of the solidified shell at the mold outlet, which show a symmetrical pattern in both the X and Y directions with different fillet radiuses. Specifically, the temperatures are around 1594–1605 K at the center of the surface (X = 0 m or Y = 0 m). As the surface temperatures gradually decrease from X = 0 m (Y = 0 m) to the region of X = 0.05 m (Y = 0.05 m) (approaching the corner), the reduction amplitude is about 20–45 K. From X = 0.05 mm (Y = 0.05 m) to the corner, the surface temperatures drop rapidly and reach the lowest value at the corner. Increasing the fillet radius from R = 6 mm to R = 25 mm raises the minimum temperature from 1376 K to 1519 K, resulting in a 10.39% increase and a significant improvement in the circumferential heat transfer uniformity of the mold.

To further investigate the impact of the fillet radius on the corner temperature, we calculated the temperature increase ratio by comparing the lowest temperature at adjacent corners with different fillet radiuses, using Formula (11). Our calculations reveal that the temperature increase ratios at the corners are 0.73%/mm, 1.15%/mm, 0.66%/mm, 0.28%/mm and 0.31%/mm when the fillet radius is R = 8 mm, 10 mm, 15 mm, 20 mm and 25 mm, respectively. Overall, the temperature increase ratio initially increases and then decreases, with a maximum value of 1.15%/mm at a fillet radius of R = 10 mm and a corresponding lowest corner temperature of 1428 K. Based on these results, increasing the fillet radius will lead to a continuous temperature rise, while the effect will gradually decrease, and the billet loss caused by an excessive fillet radius will significantly increase. Based on the comprehensive analysis, the appropriate fillet radius is around R = 10 mm, provided that the high-speed billet continuous casting mold can achieve uniform heat transfer.

(11) $$\zeta =\frac{T_{R_{l\arg e}}-T_{R_{\mathrm{small}}}}{T_{R_{\mathrm{small}}}\cdot (R_{l\arg e}-R_{\mathrm{small}})}\times 100\%$$

where ζ is the temperature increase ratio of the corner. $R_{l\arg e}$ and $R_{small}$ are the adjacent large and small fillet radiuses, respectively. $T_{R_{l\arg e}}$ and $T_{R_{\mathrm{small}}}$ are the lowest surface temperatures of the corner corresponding to $R_{l\arg e}$ and $R_{small}$.

## 4. Conclusions

(1) Compared with the conventional casting speed of 3.0 m/min, the surface temperature of the billet at the mold outlet increases by about 23.90% and the solidified shell thickness decreases by 50.66% under the high casting speed of 6.5 m/min. Therefore, improving the heat transfer capacity of the mold zone is the basis for achieving the high-speed continuous casting of billets.
(2) Increasing the effective length of a mold is the most effective way to enhance its heat transfer capability and achieve the high-speed continuous casting of billets. By extending the effective length from 900 mm to 1400 mm, the total heat increase from 12.18 MJ to 27.17 MJ and the thickness of the solidified shell at the center of the mold outlet increases from 5.85 mm to 9.15 mm, and for every 100 mm extension, the average increases are 19% and 9.21%, respectively.
(3) The thickness of the copper tube, velocity of cooling water and width of the water gap have little effect on the heat transfer capability of the billet’s high-speed continuous casting mold. However, the latter two factors have a significant impact on the increase in the temperature of the cooling water. An increase in velocity from 8 m/s to 20 m/s results in a 58.63% decrease in temperature rise, while a decrease in width from W = 5 mm to W = 3 mm leads to a 67.15% increase in temperature rise. This information is crucial in preventing the boiling of the cooling water inside the mold.
(4) Increasing the fillet radius of the mold can improve the uniformity of heat transfer in the billet’s high-speed continuous casting mold region. Considering both the uniformity of heat transfer and the loss of the billet, the optimal fillet radius for the mold is around R = 10 mm.

The transformation of theoretical research into industrial applications is a systematic project that involves various aspects such as steel ladle technology, tundish technology, mold technology and secondary-cooling technology. The significance of this paper lies in its study of the critical technology of the core component (mold) of a caster, which lays the essential theoretical and technical foundation for the industrialization of the high-speed continuous casting of billets. Further improvements to the related technologies mentioned will be made in subsequent work.

## Figures and Tables

**Table 1:** Parameters of billet’s mold region.

Parameters	Value
Cross-section size/mm × mm	160 × 160
Inner diameter of submerged entry nozzle (SEN)/mm	40
Outer diameter of SEN/mm	90
Effective mold length (L)/mm	900
Clearance height of mold/mm	100
Corner radius (R)/mm	10
Thickness of copper plate (T)/mm	14
Width of water tube (W)/mm	5
Velocity of cooling water, m/s	12
Pouring temperature, K	1808
Temperature of cooling water, K	300

**Table 2:** Physical properties of Q235 steel.

Parameters	Value
Liquidus temperature/K	1793
Solidus temperature/K	1748
Viscosity/kg·(m·s)^−1^	0.0062
Density/kg·m^−3^	7200
Specific heat/J·(kg·K)^−1^	720
Thermal conductivity/W·(m·K)^−1^	46
Latent heat of solidification/J·kg^−1^	264,000

**Table 3:** Thermal properties of materials in mathematical model.

Material	Parameters	Value
Water	Density/kg·m^−3^	998
	Specific heat/J·(kg·K)^−1^	4187
	Thermal conductivity/W·(m·K)^−1^	0.579
	Viscosity/kg·(m·s)^−1^	0.001003
Copper [17]	Density/kg·m^−3^	8940
	Specific heat/J·(kg·K)^−1^	410
	Thermal conductivity/W·(m·K)^−1^	335 (298 K)
		315 (393 K)
		310 (623 K)

**Table 4:** Values of copper tube thickness and water gap width.

Parameters	Value					
Thickness of copper tube (T), mm	8	10	12	14	14	14
width of water gap (W), mm	5	5	5	5	4	3

## Data Availability

Not applicable.
